# Polyphenols regulate bone health through the gut-bone axis: molecular and applications in animal nutrition

**DOI:** 10.1186/s40104-026-01505-9

**Published:** 2026-08-25

**Authors:** Bo Gu, Jiaqi Wang, Xiaxin Liu, Jiantao Zhang, Tianwen Ma, Baoming Shi

**Affiliations:** 1 Heilongjiang Provincial Key Laboratory of Pathogenic Mechanism for Animal Disease and Comparative Medicine, College of Veterinary Medicine, Northeast Agricultural University, Harbin, Heilongjiang 150030 China; 2 College of Animal Science and Technology, Northeast Agricultural University, Harbin, Heilongjiang 150030 China

**Keywords:** Animal nutrition, Bone health, Gut-bone axis, Gut microbiota, Polyphenols

## Abstract

This review summarizes the regulatory roles of polyphenolic compounds in animal skeletal health through the gut-bone axis and explores their benefits for animal nutrition. First, this review focuses on their key roles in maintaining skeletal homeostasis through gut microbiota, intestinal barrier integrity, immune regulation, microbial metabolites, and endocrine signaling. Then, from a biological mechanism perspective, we summarize how polyphenolic compounds regulate animal bone metabolism by reshaping gut microbiota composition, promoting short-chain fatty acid production, enhancing intestinal barrier function, reducing oxidative stress and systemic inflammation, and modulating osteogenic and osteoclastic signaling pathways. Furthermore, this review comprehensively discusses representative subclasses of polyphenolic compounds (including flavonoids, phenolic acids, lignans, stilbenes, and other bioactive compounds) and elucidates their mechanisms in animal skeletal protection and nutritional regulation. In conclusion, this review provides a theoretical foundation for understanding the polyphenol-mediated gut-bone axis regulatory mechanisms and offers new insights into nutritional strategies for promoting bone health in animals.

**Graphical Abstract:**

**Figure:**
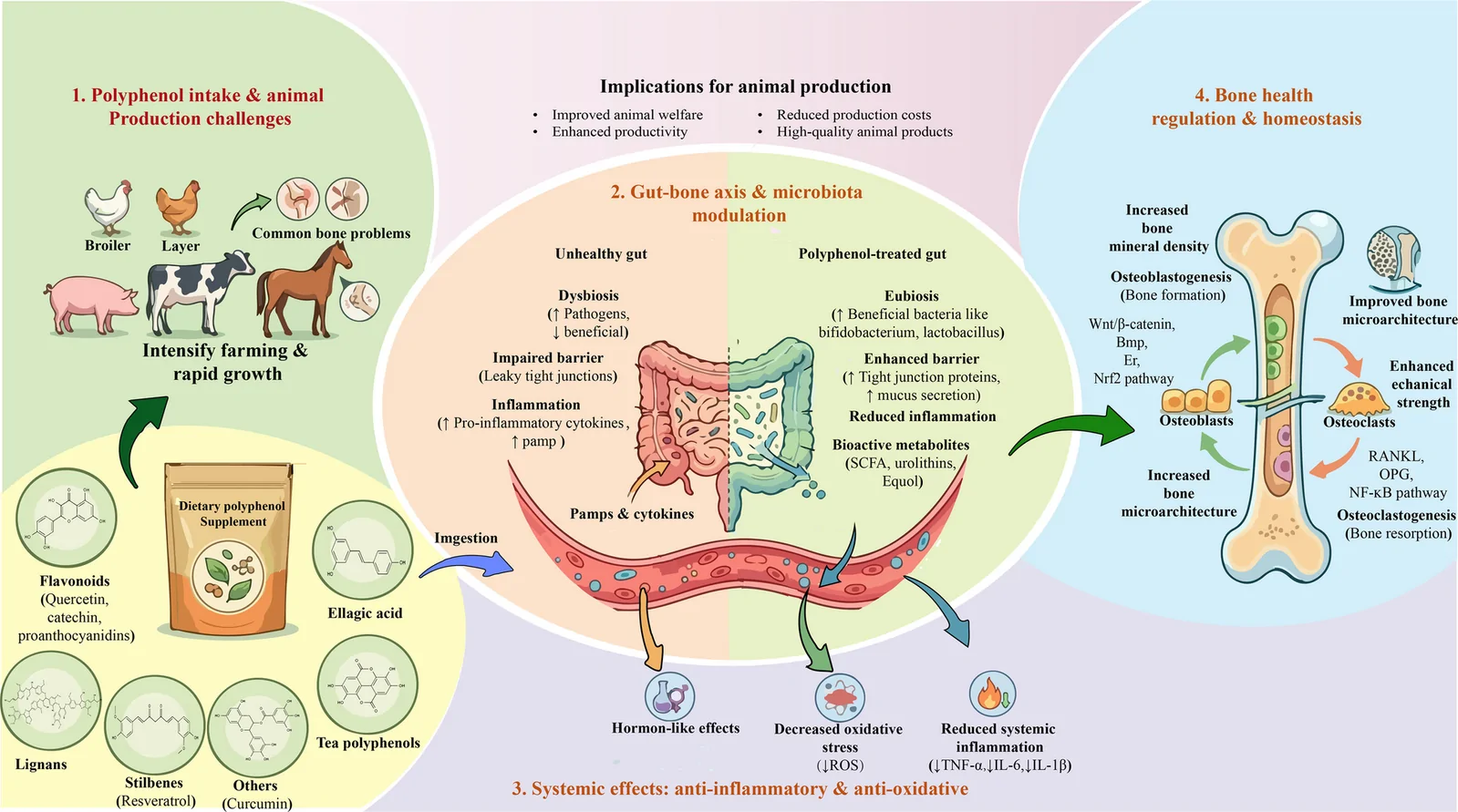

## Introduction

As the global demand for animal-derived foods continues to rise, modern livestock production has shifted toward highly intensive and precision-based systems. However, in the pursuit of high daily weight gain and feed conversion efficiency under intensive production, the rapid deposition of soft tissues often outpaces the rate of bone mineralization [1]. Under intensive farming conditions, factors such as restricted movement, altered light cycles, and high stocking density further contribute to metabolic disturbances [2]. This contradiction is particularly prominent in skeletal diseases of fast-growing broilers, degenerative joint diseases in horses and dairy cows, limb and hoof injuries in high-yielding sows, and osteoporosis in late-laying hens [3]. Bone health problems not only impair animal welfare but also directly lead to reduced feed intake, increased culling rates, and compromised carcass quality [4].

Polyphenols, a class of plant secondary metabolites, have attracted considerable attention due to their potent anti-inflammatory, antioxidant, and multi-target regulatory activities. They are natural organic compounds containing multiple phenolic hydroxyl groups and are widely distributed in virtually all plant parts, including fruits, flowers, leaves, and epidermis, exhibiting diverse bioactivities [5]. Polyphenols are traditionally categorized into five major classes: flavonoids, phenolic acids, lignans, stilbenes, and others, with over 8,000 known structures that display remarkable structural diversity [6]. Polyphenols commonly found in the Mediterranean diet have been reported to exert beneficial effects on bone health by regulating oxidative stress, inflammation, and bone remodeling [7]. Recent research has highlighted the critical role of the gut-bone axis in preserving skeletal homeostasis [8]. Polyphenols play a key role in maintaining bone homeostasis by inhibiting systemic inflammation, alleviating oxidative stress, and directly regulating the balance between osteoblasts and osteoclasts [9]. Because of their complex molecular structures, the vast majority of polyphenols reach the large intestine intact, where they are transformed by the gut microbiota (e.g., *Bifidobacterium*, *Lactobacillus*) into secondary metabolites with higher bioavailability [10]. These metabolites not only enhance the absorption of calcium, phosphorus, and vitamin D by strengthening the intestinal biological barrier, optimizing microbial composition, and improving intestinal enzyme activity, but can also enter the blood circulation and indirectly modulate the bone tissue microenvironment [11].

This review aims to evaluate the potential of polyphenols as functional feed additives to improve animal bone health by modulating the gut microbiota and its metabolites. By integrating research in animal production, we summarize recent advances regarding the mechanisms of action and related advancements in the relationship between polyphenols and animal nutrition.

## Literature search strategy

This narrative review was based on a literature search of PubMed, Web of Science, and Scopus databases. Relevant studies published between approximately 2000 and 2026 were identified using combinations of keywords related to “polyphenols”, “flavonoids”, “phenolic compounds”, “bone health”, “bone metabolism”, “osteoblast”, “osteoclast”, “osteoporosis”, “gut microbiota”, and “gut-bone axis”. Additional studies were identified by screening reference lists of key articles and recent reviews. Studies were selected based on their relevance to polyphenol-mediated regulation of bone health, underlying mechanisms, and applications in animal production. Evidence from livestock species was prioritized to evaluate practical applications, while rodent, cellular, and human studies were included to provide complementary mechanistic and translational insights when direct evidence from production animals was limited.

## Polyphenol-driven remodeling of the gut-bone axis: biological mechanisms

### Polyphenols trigger the initiation of gut-bone axis signaling

**Fig. 1:**
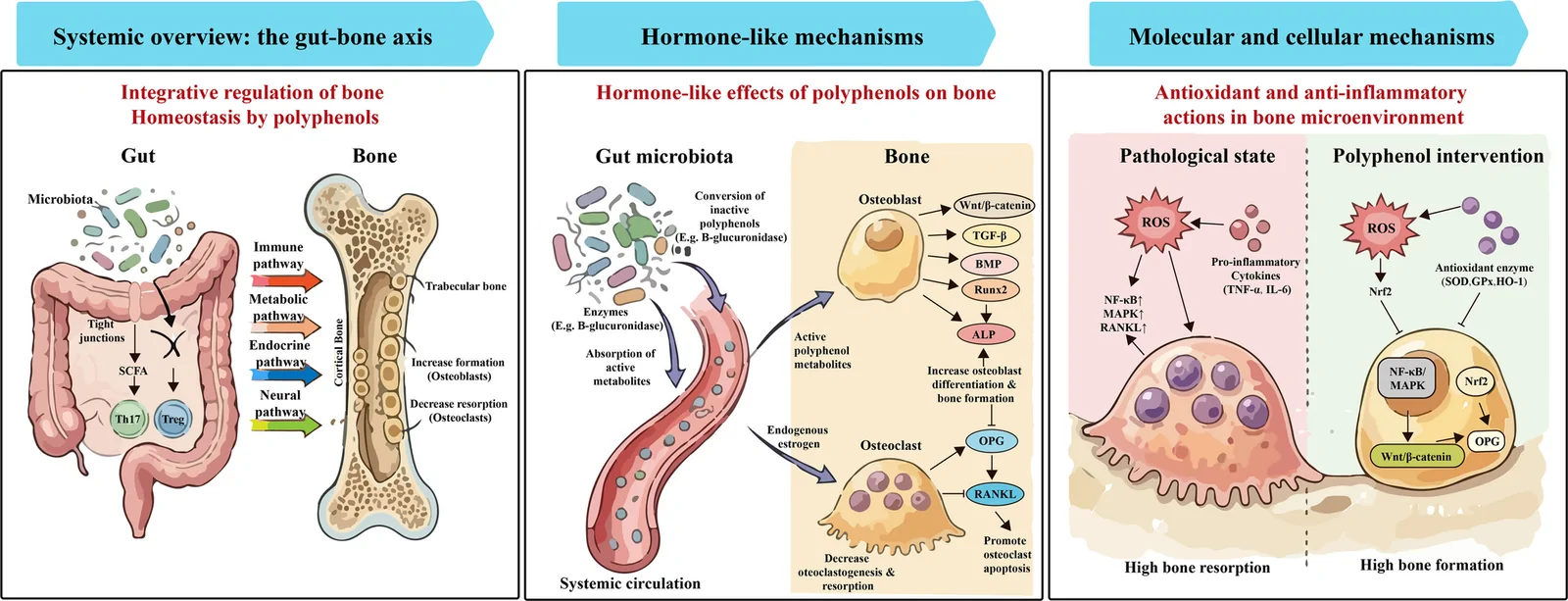
Proposed mechanisms by which polyphenols maintain bone homeostasis through the gut-bone axis. Polyphenols regulate bone metabolism by remodeling gut microbiota, enhancing intestinal barrier function, modulating immune and metabolic communication, and promoting osteoblastogenesis while suppressing osteoclastogenesis. In addition, polyphenols alleviate oxidative stress and inflammation in the bone microenvironment through antioxidant and anti-inflammatory pathways, thereby maintaining skeletal homeostasis

In intensive production systems, livestock often face conflicts between rapid growth and insufficient skeletal development, leading to animal skeletal disorders that cause severe locomotor dysfunction, high culling rates, and carcass condemnation (Table 1). Through immunity, metabolism, endocrine, and neural pathways, the gut microbiota finely regulates the activities of osteoclasts and osteoblasts, controls nutrient absorption, hormone levels, and inflammatory status, and constructs a gut-bone axis that serves as a crucial hub for controlling bone health [12]. When gut microbiota homeostasis is disrupted, the expression of tight junction proteins between epithelial cells is downregulated, leading to increased intestinal permeability [13]. Pro-inflammatory antigens such as lipopolysaccharide (LPS) can enter the systemic circulation, triggering metabolic endotoxemia [14]. Antigens trigger the secretion of large amounts of pro-inflammatory cytokines, which further act on osteoblasts and osteoclasts, inhibiting bone formation and enhancing bone resorption by regulating pathways such as receptor activator of nuclear factor kappa-B ligand (RANKL), Wnt, nuclear factor kappa B (NF-κB), and mitogen-activated protein kinase (MAPK) [15]. In addition, gut microbiota dysbiosis affects the endocrine system, leading to abnormal calcium absorption and reduced bioavailability of vitamins D and K, thereby reducing bone strength in animals from a nutritional metabolism perspective [16]. Polyphenol effects show multi-level synergistic characteristics, including strengthening the physical barrier, inhibiting oxidative stress and inflammation, and modulating gut microbiota composition [17]. First, polyphenols can directly upregulate the expression of tight junction proteins and stimulate mucus secretion by goblet cells in the intestinal wall, enhancing the physical barrier function and blocking the trans-epithelial transport of harmful substances. Second, polyphenols have prebiotic-like effects and have also been shown to accelerate epithelial cell migration and proliferation, promoting intestinal barrier repair [18]. Finally, by enhancing gut barrier function, polyphenols inhibit damage caused by harmful substances, promote the absorption of bioactive metabolites into the systemic circulation, and modulate immune cell function, thereby contributing to bone health maintenance [19]. In addition, polyphenols reduce the levels of circulating inflammatory factors by repairing the intestinal barrier [20]. In summary, polyphenols effectively lower systemic inflammation levels by enhancing intestinal barrier integrity, thereby alleviating inflammatory factor-mediated bone damage (Fig. 1).

**Table 1:** Common bone problems in farm animals

Species	Common bone-related diseases
Broilers	Tibial dyschondroplasia, femoral head necrosis/antemortem spinal cord displacement, cage layer fatigue
Laying hens	Osteopetrosis, cage layer fatigue
Dairy cows	Parturient paresis, laminitis
Pigs	Leg weakness syndrome, osteochondrosis
Horses	Osteoarthritis, sesamoiditis, osteoporosis/fibrous osteodystrophy, osteochondrosis

### Polyphenols maintain bone metabolic homeostasis via synergistic antioxidant and anti-inflammatory mechanisms

Excessive accumulation of reactive oxygen species (ROS) can cause damage to bone cells and compromise bone tissue integrity [21]. Under the stress induced by intensive farming, excessive accumulation of ROS causes protein denaturation, lipid peroxidation, and DNA damage, and, through the release of pro-inflammatory cytokines, pushes the bone microenvironment toward a pathological state favoring bone resorption [22]. Oxidative stress also leads to abnormalities in intestinal structure and function, disrupts the microbiota, and impairs nutrient absorption and utilization, ultimately affecting animal health [23].

**Fig. 2:**
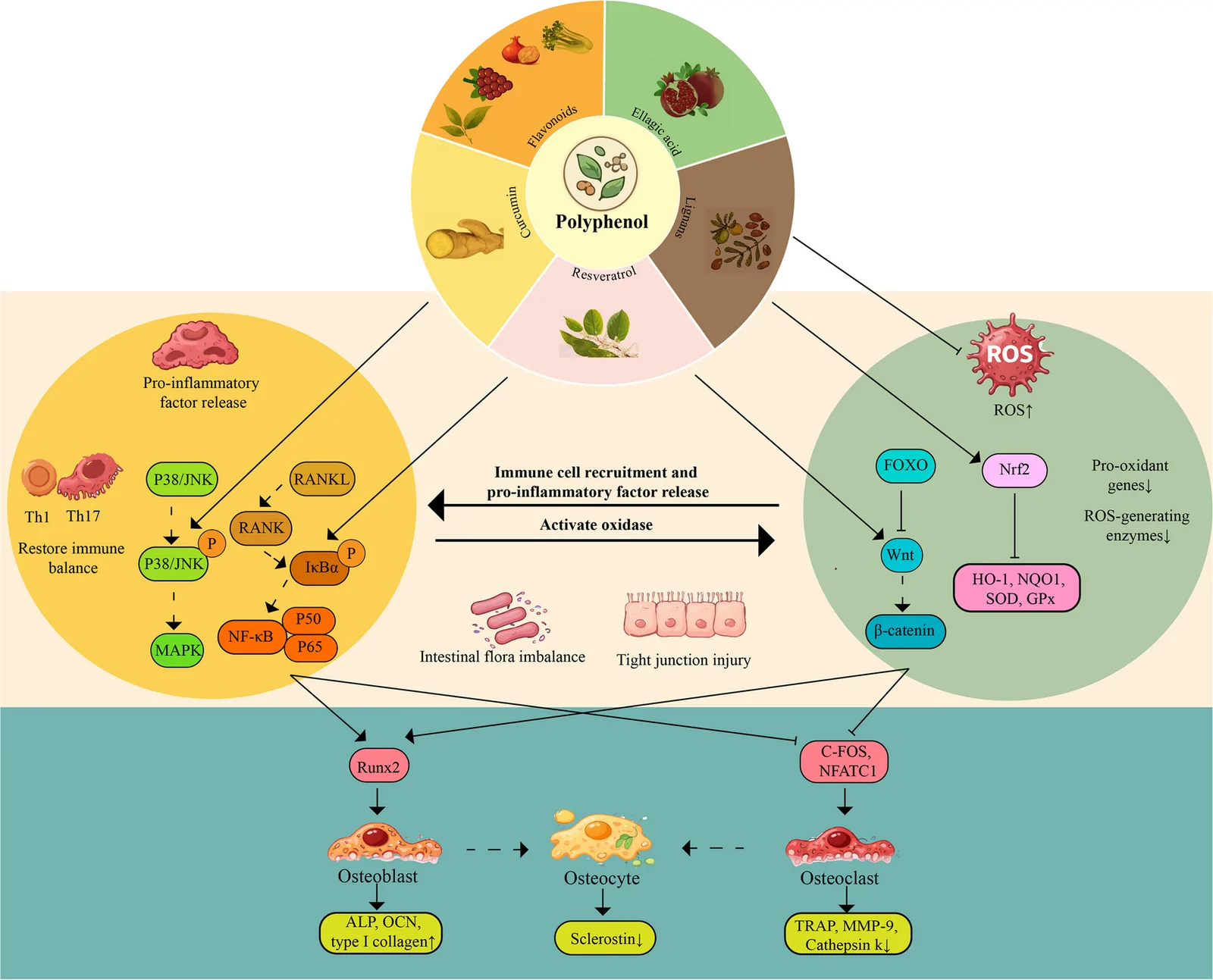
Molecular mechanisms by which polyphenols maintain bone metabolic homeostasis through antioxidant and anti-inflammatory regulation. Polyphenols alleviate gut microbiota dysbiosis, intestinal barrier disruption, and inflammatory responses by modulating NF-κB, MAPK, and RANKL/RANK signaling pathways. Meanwhile, polyphenols activate antioxidant pathways, including Nrf2/HO-1 and Wnt/β-catenin signaling, to enhance antioxidant defense, regulate osteoblast and osteoclast activity, and maintain bone remodeling homeostasis

Polyphenols, with their characteristic array of phenolic hydroxyl groups, exhibit outstanding dual properties of inhibiting oxidative stress and resisting inflammation (Fig. 2). First, as natural reducing agents, polyphenols can directly donate hydrogen atoms to neutralize ROS, reducing oxidative stress levels [24]. Second, polyphenols activate the nuclear factor erythroid 2-related factor 2 (Nrf2) signaling pathway, promoting the transcription of antioxidant genes, including heme oxygenase-1 (*HO-1*), NAD(P)H quinone oxidoreductase 1, and superoxide dismutase (*SOD*), thereby enhancing antioxidant defense and maintaining redox homeostasis [25]. At the immune regulation level, polyphenols and short-chain fatty acids (SCFAs) block the phosphorylation of IκBα, p38, and JNK to inhibit inflammatory signaling pathways such as NF-κB and MAPK, thereby maintaining the immune balance between Th1/Th17 and Treg cells and downregulating NLR family pyrin domain-containing protein 3 (NLRP3) inflammasome activation [26]. By acting at the molecular level on osteoblasts, osteocytes, or osteoclasts and by modulating pathways such as Wnt/β-catenin, NF-κB, MAPK, RANKL-receptor activator of nuclear factor kappa-B (RANK), and Nrf2, polyphenols not only promote osteoblast differentiation but also effectively curb osteoclastic destruction, thereby achieving remodeling of the bone microenvironment and restoration of homeostasis within the complex framework of the gut-bone axis [27].

### Polyphenols exert hormone-like effects and endocrine regulation in bone remodeling

**Fig. 3:**
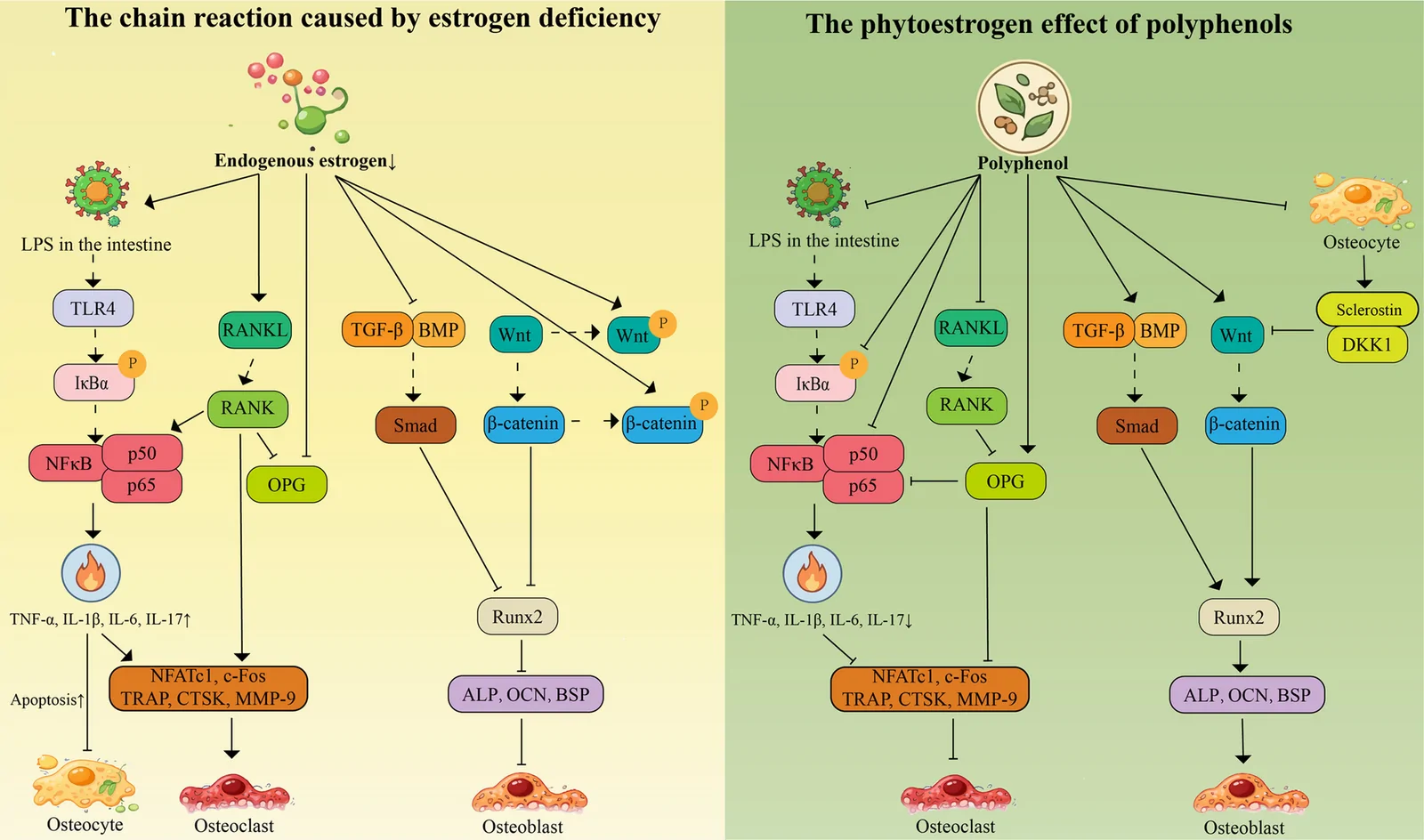
Phytoestrogen-like effects of polyphenols on bone remodeling under estrogen-deficient conditions. Estrogen deficiency promotes inflammatory responses and RANKL/RANK-mediated osteoclastogenesis while impairing osteogenic signaling. Polyphenols partially mimic estrogen activity by reducing inflammation, enhancing OPG expression, inhibiting osteoclast differentiation, and promoting osteoblastogenesis through Wnt/β-catenin, TGF-β/BMP, and Runx2-related pathways. Polyphenols also suppress osteocyte-derived sclerostin and DKK1, thereby maintaining bone remodeling homeostasis

Some polyphenols are classified as phytoestrogens because their structures resemble endogenous estrogens (such as isoflavone, lignan, resveratrol). After being absorbed into the systemic circulation via the gut-bone axis, these active molecules can act as ligands to bind estrogen receptors (ERα/ERβ) on intestinal and bone cells, thus reversing a series of adverse effects caused by hormonal fluctuations (such as reproductive decline in breeding stock and the late laying phase of high-producing hens) [28]. In the bone microenvironment, polyphenols, by mimicking estrogen, bind to ERs highly expressed on osteoblasts, osteoclasts, and osteocytes, initiating a series of protective signaling cascades (Fig. 3). The polyphenol-receptor complex can activate classical pathways such as Wnt/β-catenin, TGF-β, and bone morphogenetic proteins (BMPs), promoting bone matrix synthesis and mineralization [29]. Polyphenols regulate the RANKL/RANK/osteoprotegerin (OPG) axis, promoting high expression of OPG, which competitively blocks RANKL binding to RANK, thereby reducing bone resorption [30]. Additionally, the estrogen-mimetic effect can increase TGF-β production, inducing osteoclast apoptosis [31]. Polyphenols can effectively modulate sclerostin synthesis in osteocytes, inhibiting the Wnt pathway and thus increasing bone formation, to maintain skeletal structural stability under high-intensity production conditions [32]. Polyphenols, with estrogenic activity, can intervene in the nervous and endocrine systems, promoting the secretion and metabolism of reproductive and growth-related hormones [33, 34].

## The potential of different types of polyphenols to regulate animal nutrition and bone health

### Flavonoids

Quercetin, a typical representative of flavonols, can optimize the gut microbiota through both direct bacteriostatic and prebiotic effects [35]. Quercetin directly inhibits harmful bacteria (e.g., *Escherichia coli*,* Salmonella*, and *Clostridium*) by disrupting biofilm formation and downregulating the expression of adhesin and toxin genes, thereby reducing their invasiveness. In addition, quercetin specifically promotes the growth of beneficial bacteria (such as *Bifidobacterium*,* Lactobacillus*, and *Akkermansia*), restoring microbial diversity and enhancing stability and resilience [36]. In the intestines of aged breeder chickens, quercetin modulates the activity of apoptosis-related genes, inhibits the expression of inflammatory cytokines, promotes the correct anchoring of tight junction proteins on the cell membrane, reduces epithelial cell apoptosis, and restores barrier integrity [37]. Quercetin stabilizes NRF2 protein, enhances cellular antioxidant capacity and protects epithelial cells from oxidative damage [38]. Quercetin increases the expression of osteoblast-specific genes and suppresses osteoclast markers, promoting bone synthesis [39]. After quercetin intake, broilers showed decreased malondialdehyde (MDA) levels and increased SOD levels, along with enhanced antioxidant activity. This study proved that by improving the gut microbiota, quercetin achieved a positive effect of reducing the risk of metabolic diseases and improving growth performance and yield indicators [40]. A similar study showed that by remodeling gut microbiota structure, quercetin enhanced the mRNA expression of nutrient transporters such as glucose transporter type 2 (GLUT2), peptide transporter 1 (PEPT1), and fatty acid synthase (FAS) in the intestine, promoting broiler growth and development [41]. After supplementation with quercetin and its derivatives, the relative abundance of *Agathobacter* and *Alloprevotella* increased in the piglet intestine, further influencing downstream amino acid metabolism and absorption, ultimately improving piglet growth performance [42]. One study involving laying hens fed quercetin demonstrated that quercetin significantly increased femoral mineral content and density, improved bone microstructure, and regulated the balance of bone remodeling [43]. Another study in laying hens showed that dietary supplementation with quercetin increased antioxidant capacity, improved cecal microbiota structure, and significantly elevated levels of production-related hormones such as estradiol (E2), luteinizing hormone (LH), follicle-stimulating hormone (FSH), and growth hormone (GH), thereby improving laying performance [34]. In conclusion, quercetin primarily protects animal health and promotes production performance by regulating systemic inflammation through gut microbiota and metabolites.

Apigenin is widely found in plant-based feed sources such as celery, parsley, and pistachios. Apigenin can activate Wnt/β-catenin to promote osteogenic differentiation of mesenchymal stem cells and regulate bone metabolism [44]. Under inflammatory or oxidative stress conditions, quercetin and apigenin can act on macrophages, inhibiting excessive activation of the MAPK pathway by preventing p38 and JNK subtype activation, thereby alleviating inflammation-induced skeletal damage [45–47]. Studies indicate that dietary apigenin can counteract the damage of enteritis on broiler health and improve growth performance by reducing oxidative stress, regulating inflammation, improving intestinal barrier function, and inhibiting the proliferation of harmful gut bacteria [48]. In addition, apigenin can significantly improve the egg production rate and egg quality of laying hens by inhibiting intestinal immune responses, and increase feed intake, daily weight gain and feed conversion ratio in piglets [49, 50]. Overall, apigenin plays multiple biological roles in promoting bone health and improving animal production performance by synergistically regulating intestinal homeostasis, inflammation and oxidative stress responses, and bone metabolism-related signaling pathways.

Proanthocyanidins (PACs), as polymeric polyphenols widely present in grapes, cocoa, and leaves, have attracted attention for their antiviral, antibacterial, and antioxidant activities [51]. Research has shown that dietary PACs supplementation in Kangle chickens can improve intestinal redox homeostasis by directly scavenging intestinal ROS, activating the Nrf2 antioxidant system [52]. Appropriate PACs intake helps improve intestinal mucosal structure in weaned piglets, reduces intestinal permeability, and promotes nutrient absorption to improve production performance [53–55]. Studies on the addition of PACs to broiler diets have found that PACs improve growth performance by regulating gut microbiota structure, increasing SCFA levels, inhibiting inflammation, and influencing amino acid and lipid metabolism [56]. Similar changes were observed with dietary PACs in geese [57]. At the cellular signaling level, PACs effectively inhibit NF-κB and MAPK pathways by blocking the phosphorylation of IκBα and JNK subunits, thereby significantly suppressing osteoclast differentiation and bone resorption activity [58]. By strengthening intestinal physical protection, optimizing microbiota structure, and intervening in systemic metabolism, proanthocyanidins construct a comprehensive protection system from the gut to the bone.

Tea polyphenols (TPs), also known as tea tannins, are a collection of polyphenols primarily composed of catechins, flavonoids, and other flavonoid compounds. TPs are recognized as highly effective natural antioxidants due to their excellent ROS scavenging ability, metal ion chelating activity, and precise regulation of redox signaling pathways (Nrf2, NF-κB, MAPK, etc.) [59]. In nutritional studies on goat kids, the addition of TPs significantly inhibited excessive intestinal ROS production, systematically strengthening the intestinal immune barrier and maintaining microbial homeostasis by downregulating the toll-like receptor 4 (TLR4)/NF-κB signaling axis and the expression of downstream inflammatory cytokines [60]. Dietary supplementation with TPs restored the hens’ antioxidant capacity and microbiota homeostasis, increased egg production rate and yolk color, and reversed the damage caused by molybdenum [61]. Research has shown that TPs can remodel the gut microbiota of squabs, increase SCFA levels, restore intestinal integrity, promote intestinal villus development to improve nutrient absorption, enhance antioxidant capacity, and reduce inflammation [62]. Additionally, TPs significantly decreased lipase and amylase activities, promoted lipid oxidation and excretion, and reduced lipid metabolism [62]. The combined use of TPs and probiotics has become a popular choice for green feed supplementation. Multiple studies have confirmed that dietary TP and probiotic supplementation significantly increase antioxidant capacity, regulate intestinal barrier function and microbiota structure, enhance immune levels, and ultimately improve the health performance of broilers and laying hens [63–65].

### Phenolic acids

Ellagic acid (EA) is a natural polyphenol widely found in plants such as strawberries, raspberries, and pomegranates, possessing diverse biological activities, including antioxidant, anticancer, and antimutagenic properties [66]. After being metabolized by the gut microbiota into urolithins, EA specifically accumulates in animal bones, promotes calcium ion absorption during bone mineralization, influences bone formation and regulates bone health. Studies on 1-year-old thoroughbred horses supplemented with EA showed improved antioxidant capacity and gut microbiota diversity [67]. Dietary supplementation with EA in piglets also significantly alleviated intestinal injury, dysbiosis, and oxidative stress, increased SCFA levels, and thereby improved growth performance [68]. The addition of EA to feed can alter the gut microbiota structure of lactating mares, stimulate the production of SCFAs, enhance antioxidant capacity, and improve milk yield and quality [69]. EA can enhance the antioxidant capacity and intestinal barrier function of heat-stressed broilers by regulating the gut microbiota, thereby promoting carbohydrate and lipid metabolism and improving broiler production performance [70]. EA can not only achieve systemic metabolic regulation by optimizing the gut environment but also provide precise protection to the bone tissue microenvironment by directly intervening with key signaling molecules.

### Lignans

Lignans are complex phenolic macromolecules with antioxidant and antibacterial bioactivity, and widely present in flaxseed and sesame [71]. The addition of polyphenols rich in lignans to piglet diets can improve the gut microbiota, reduce the production of reactive oxygen species and inflammatory cytokines in small intestinal epithelial cells, increase intestinal butyrate levels, and enhance intestinal barrier function [72]. Precise intervention in the serotonin pathway of the gut-bone axis is regarded as a core mechanism by which lignan protects bone health. Research has indicated that lignans and their gut metabolites can directly bind tryptophan hydroxylase 1 (TPH-1), thereby specifically inhibiting intestinal serotonin synthesis [73]. Gut-derived serotonin is transported to bone through the bloodstream, where it acts on the 5-HT1b receptor in preosteoblasts and activates the activating transcription factor 4 (ATF4)/forkhead box O1 (FOXO1) complex in bone, which inhibits osteoblast proliferation and consequently reduces bone formation. After lignan lowers serotonin levels, the binding of FOXO1 to ATF4 is decreased, and bone formation is restored. In summary, lignans regulate the tryptophan-serotonin pathway in the gut-bone axis to promote bone formation, increase bone density, and exert anti-osteoporosis effects [73].

### Stilbenes

Resveratrol (RES) is the most representative active component of the stilbene class, widely found in grape skins, peanuts, and *Polygonum cuspidatum*. At the bone tissue microenvironment level, RES exerts a dual regulatory role. As a natural ligand, RES activates ER in bone cells, and through the ER-dependent MAPK pathway and the osteogenesis-mediating Wnt/β-catenin pathway, drives bone marrow mesenchymal stem cell (BMSC) proliferation and osteoblast differentiation, significantly enhancing bone mineralization and calcium deposition [74]. Clinical studies have confirmed that RES can downregulate the bone resorption marker catalase (CTX) and increase bone mineral density (BMD) [75]. At the same time, RES activates the Nrf2/HO-1 axis, enhancing endogenous antioxidant enzyme activity to protect bone cells from oxidative damage by ROS and antagonizing the destructive effects of inflammatory cytokines [76]. In terms of animal production performance, RES can inhibit intestinal oxidative damage and inflammation by regulating gut microbiota structure, enhancing SCFAs, and activating the Nrf2 antioxidant system, thus playing a protective role in weaned piglets and stressed broilers [77, 78]. Simultaneously, RES improves the growth performance of early-weaned calves through its anti-inflammatory and antioxidant effects, regulation of gut microbiota, and tryptophan metabolism [79]. In summary, RES shows great promise in improving animal production performance and preventing bone damage caused by metabolism or stress, making it a valuable functional feed additive.

### Others

Curcumin, a representative diarylheptanoid compound mainly enriched in *Curcuma longa*, has attracted considerable attention due to its properties of combating oxidative stress and improving gut microbiota [80, 81]. The hypothesis that curcumin exerts bone-protective effects by remodeling the gut microbiota has been widely validated. Experiments have demonstrated that curcumin can reduce bone loss and restore BMD [82]. In livestock production practice, the addition of curcumin nanoparticles to the diet of calves under heat stress significantly alleviate the negative effects of heat stress on skeletal development, effectively safeguarding the skeletal growth performance of young livestock [83]. Regarding the molecular mechanism of bone protection, curcumin can regulate the gut microbiota, increase the abundance of *Akkermansia muciniphila* and *Dubosiella*, reduce intestinal barrier damage and systemic inflammation by interfering with the arachidonic acid metabolism pathway, inhibit bone resorption, promote bone formation, and exert a bone-protective effect [82]. Other studies have shown that dietary curcumin supplementation, by regulating cecal microbiota and its metabolites, enhances the intestinal barrier function in broilers, inhibits inflammation, influences lipid metabolism, and ultimately promotes broiler growth performance [84]. Dietary curcumin accumulates in the yolk in a dose-dependent manner and transgenerationally enhances the antioxidant capacity of offspring chicks [85]. Curcumin exerts its bone-protective effects by regulating gut microbiota and antioxidant pathways.

**Fig. 4:**
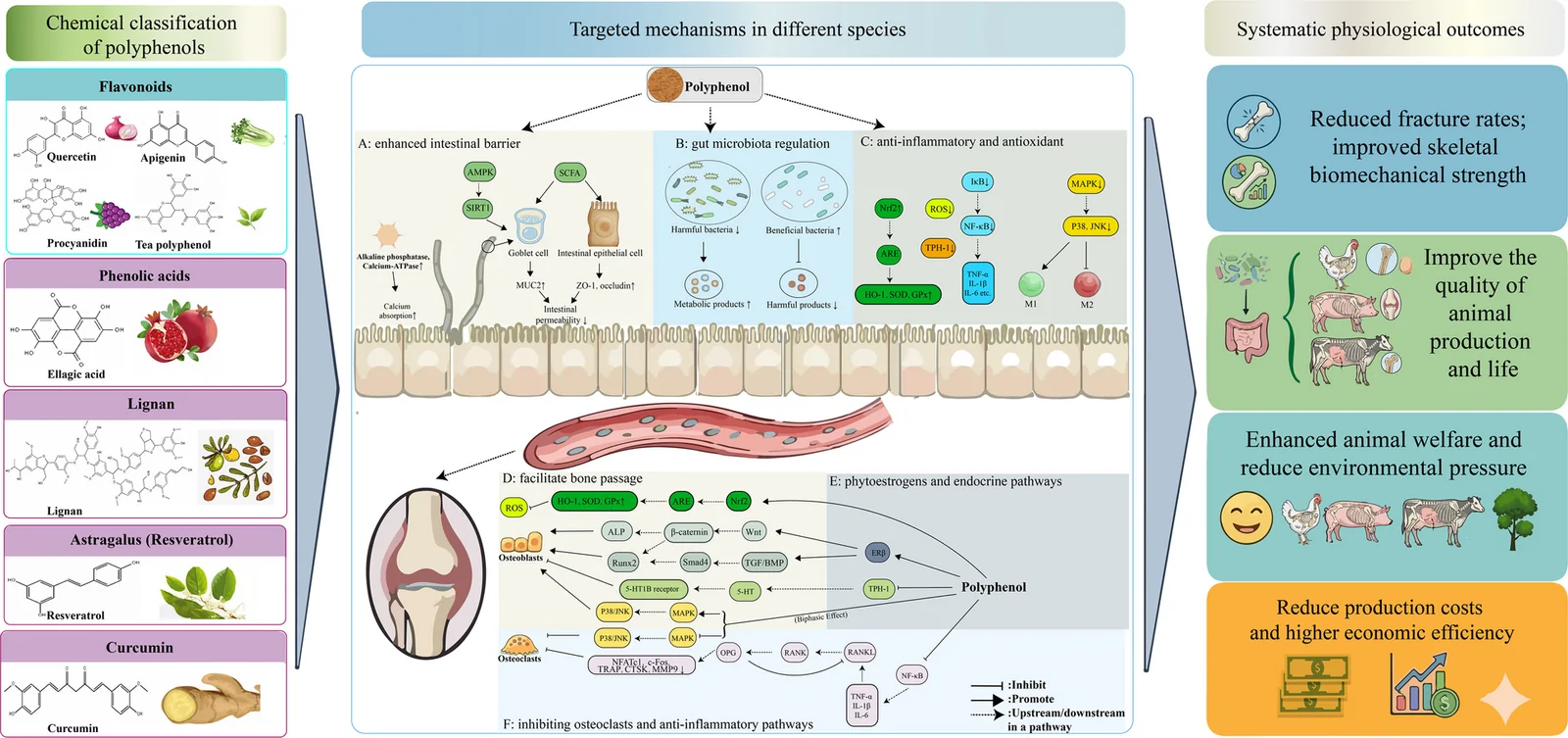
Integrated framework illustrating the classification of polyphenols, their multi-target regulatory mechanisms, and their potential applications in improving bone health in animals. Major polyphenol classes, including flavonoids, phenolic acids, stilbenes, and curcuminoids, regulate bone homeostasis through coordinated actions on gut microbiota, intestinal barrier function, oxidative stress, inflammation, and endocrine-related pathways. These multi-target effects promote osteoblast activity, suppress osteoclastogenesis, and contribute to improved skeletal health, animal welfare, and sustainable livestock production

In summary, the molecular mechanisms and a summary of how different types of polyphenols regulate animal nutrition and bone health are presented in Table 2 and Fig. 4. It is noteworthy that most studies on polyphenols have not addressed their bioavailability levels. Therefore, differences in bioavailability may partly explain the inconsistent efficacy observed among studies using different polyphenol sources, formulations, doses, and animal models. This can be primarily attributed to the following factors: polyphenol metabolites are highly diverse, structurally complex, and exhibit high potency at trace levels, making comprehensive tracking difficult with current analytical techniques. Second, after dietary intake, the bioavailability of polyphenols is strongly influenced by food source, polyphenol type, and the composition of the host gut microbiota, which makes accurate determination challenging [86]. Finally, the functional outcomes resulting from sustained polyphenol intake may depend more on cumulative signaling regulation than on plasma concentrations [87].

**Table 2:** Molecular mechanisms of polyphenols: actions, signaling pathways, and implications for animal nutrition and health

Polyphenol type	Experimental model	Administration route and dosage	Mechanism	Health effects	Closed-loop validation status	Nutrition function	References
Quercetin	Hyline brown laying hens	Purity ≥ 97%, dietary admixture, 0.05% diet, ad libitum for 10 weeks	Modulated gut microbiota (Bacteroidaceae, *Bacteroides* ↓; Lactobacillaceae, *Lactobacillus* ↑); SCFAs ↑, IL-1β and TLR-4 ↓; intestinal MDA ↓; SOD, GPx ↑; E2, LH, FSH, GH, IGF-1 ↑	Reduced oxidative stress, improved gut microbiota, and enhanced nutrient absorption	Polyphenol → intestine	/	[34]
Quercetin	Breeding hens	Purity ≥ 97%, dietary admixture, 0.4 g/kg diet, ad libitum for 14 weeks	Tight junction proteins (ZO-1, Occludin, claudin1) ↑; mucin (MUC2) ↑; Pro-inflammatory genes (*TNF-α*, *IL-6*, *IL-1β*) ↓; Anti-inflammatory genes (*IL-10*, *IL-4*) ↑; Antioxidant enzymes SOD; CAT, GPx ↑	Reduced intestinal permeability, oxidative stress, and inflammation; improved intestinal physiological function	Polyphenol → intestine	/	[37]
Quercetin	Broilers	Purity ≥ 97%, dietary admixture, 200, 500 mg/kg diet, ad libitum for 21d	Intestinal ROS, MDA ↓; LPS ↓; SOD, GPx ↑; Nrf2, HO-1 ↑; phosphorylation of ERK, JNK, and p38 ↑; Regulated the MAPK, Nrf2 pathway	Inhibited LPS-induced intestinal oxidative stress and alleviated intestinal damage	Polyphenol → intestine	/	[38]
Quercetin	Jingfen No. 6 laying hens	Purity ≥ 97%, dietary admixture, 0.4 mg/kg diet, ad libitum for 12 weeks	Tight junction proteins (Occludin, claudin1) ↑; MUC2 ↑; Modulated gut microbiota (Firmicutes ↓; Bacteroidetes ↑); SCFAs ↑; IL-1β and TLR-4 ↓; intestinal ROS, MDA ↓; SOD, GPx ↑	Alleviated intestinal inflammation and improved intestinal barrier function by modulating gut microbiota	Polyphenol → intestine	/	[39]
Quercetin	Arbor Acres broiler chickens	Purity ≥ 97%, dietary admixture, 5, 10, 15 mg/kg diet, ad libitum for 6 weeks	Leukocyte, lymphocyte ↑; Neutrophil, monocyte, and eosinophil ↓; Tight junction proteins ↑; Modulated gut microbiota (*Faecalibacterium *↑); SCFAs ↑; Intestinal ROS, MDA ↓; SOD ↑	Enhanced intestinal integrity, reduced oxidative stress, and inflammation, improved gut microbiota, and enhanced nutrient absorption	Polyphenol → intestine → growth performance	BW ↑; FCR ↓; ADG ↑	[40]
Quercetin	Ross 308 chicks	Purity ≥ 97%, dietary admixture, 200, 400, 800 mg/kg diet, ad libitum for 5 weeks	Intestinal villus height ↑; Modulated gut microbiota *Clostridium perfringens* ↓); SCFAs ↑; *GLUT2*, *PEPT1*, *FAS* mRNA ↑; Intestinal ROS, MDA ↓; SOD, GPx ↑	Reduced oxidative stress, improved gut microbiota and enhanced nutrient absorption	Polyphenol → intestine → growth performance	BW↑; FCR ↓; PER ↑	[41]
Quercetin	Hy-Line brown laying hens	Dietary admixture, 500 mg/kg diet, ad libitum for 12 weeks	ERα, ERβ, VDR ↑ (enhanced intestinal calcium absorption); Regulate the ER pathway; ALP, OPN, TRAP ↑ (early eggshell calcification, enhanced bone formation and resorption); ALP ↓, TRAP ↑ (eggshell calcification growth phase, promotes priority supply to eggshell)	Improved intestinal calcium absorption rate and calcium metabolism via estrogen-like effects. Regulated bone resorption and formation, promoted medullary bone formation, increased femoral mineral content and density, improved bone microstructure	Polyphenol → intestine → bone	/	[43]
Apigenin	White-feathered broilers	Purity ≥ 95%, dietary admixture, 125, 250, 500 mg/kg diet, ad libitum for 5 weeks	Tight junction proteins (ZO-1, Occludin, claudin1) ↑; MDA ↓; SOD, CAT ↑; IL-1β, IL-6, TNF-α ↓	Reduced intestinal permeability, oxidative stress, and inflammation; improved intestinal physiological function	Polyphenol → intestine → growth performance	ADG, ADFI ↑; FCR ↓	[48]
Proanthocyanidins	Duroc × Landrac × Yorkshire piglets	Purity ≥ 95%, dietary admixture, 50, 100, 150 mg/kg diet, ad libitum for 28 d	Modulated gut microbiota (*Fournierella*, *Oscillospira*, NK4A214_group, UCG-005↑); SCFAs ↑; MDA ↓; GPx ↑; IL-1β; IL-6; TNF-α↓	Reduced oxidative stress and inflammation. Improved gut microbiota	Polyphenol → intestine	ADG, ADFI ↑	[54]
Proanthocyanidins	Duroc × Landrac × Yorkshire piglets	Purity ≥ 95%, dietary admixture, 0, 15, 30, 60, 120 mg/kg diet, ad libitum for 28 d	Modulated gut microbiota (*Akkermansia*, *Alistipes*, *Bacteroides* ↑); SCFAs ↑; MCP-1, IL-6, TNF-α ↓	Reduced oxidative stress and inflammation. Improved lipid metabolism	Polyphenol → intestine → growth performance	ADG, ADFI ↑	[55]
Proanthocyanidins	Arbor Acres broiler chickens	Grape seed extract (Purity ≥ 80%), dietary supplementation with 200–400 mg/kg for 21d	Modulated gut microbiota (*Parasutterella*, *Lewinella*, *Lactobacillus* ↑); SCFAs ↑; MDA ↓; GPx, SOD, CAT ↑; IL-1β, IL-6, TNF-α ↓	Reduced oxidative stress and inflammation. Improved amino acid, lipid metabolism	Polyphenol → intestine → growth performance	ADG, ADFI ↑; FCR ↓	[56]
Proanthocyanidins	Sichuan white geese	Purity ≥ 95%, dietary admixture, 50, 100, 150 mg/kg diet, ad libitum for 6 weeks	Modulated gut microbiota (*Coprostanoligenes*, *Faecalibacterium* ↑); SCFAs ↑; SOD, CAT ↑	Enhanced intestinal integrity. Reduced oxidative stress, inflammation. Improved gut microbiota, amino acid metabolism	Polyphenol → intestine	/	[57]
Tea polyphenols	Leizhou goat kids	Purity ≥ 98%, dietary admixture, 2, 4, 6 g/kg diet, ad libitum for 45 d	Altered gut microbiota composition (Verrucomicrobiota, Candidatus Soleaferrea, *Prevotella* ↑); acetate (a SCFA) ↑; Serum MDA ↓; antioxidant enzymes CAT, SOD, GPx ↑; Intestinal ROS ↓; Nrf2 ↑; IL-1β, IL-6, TNF-α, IFN-γ ↓; IL-10 ↑; NF-κB, Myd88, TLR4 ↓; Regulated the TLR4/NF-κB, Nrf2 pathway	Enhanced intestinal Nrf2 antioxidant pathway and inhibited TLR4/NF-κB inflammatory pathway expression via increased gut microbiota and acetate levels, enhancing anti-inflammatory and antioxidant capacity	Polyphenol → intestine	/	[60]
Tea polyphenols	Lohmann laying hens	Purity ≥ 98%, dietary admixture, 600 mg/kg diet, ad libitum for 12 weeks	Modulated gut microbiota (*Lactobacillus agilis* ↑); MDA ↓; GPx, SOD ↑	Reduced oxidative stress. Improved gut microbiota	Polyphenol → intestine → growth performance	Increased egg production rate and egg yolk color	[61]
Tea polyphenols	White King x Shenwang pigeons	Purity ≥ 98%, dietary admixture, 100, 200, 400 mg/kg diet, ad libitum for 28 d	Intestinal villus height ↑; Modulated gut microbiota (*Lactobacillus*, *Aeriscardovia*, and *Bifidobacterium* ↑); MDA ↓; GPx, SOD ↑	Reduced oxidative stress and inflammation. Improved gut microbiota, lipid metabolism	Polyphenol → intestine → growth performance	ADG, ADFI ↑	[62]
Tea polyphenols	Hy-Line brown	Purity ≥ 98%, dietary admixture, 300 mg/kg diet, ad libitum for 8 weeks	Intestinal villus height ↑; Modulated gut microbiota (Bacteroidetes ↓); MDA ↓; GPx, SOD ↑; E2, PROG ↑	Reduced oxidative stress. Improved gut microbiota, amino acid metabolism	Polyphenol → intestine → growth performance	Increased the average egg weight, average egg mass	[64]
Tea polyphenols	Lohmann laying hens	Purity ≥ 95%, dietary admixture, 300 mg/kg diet, ad libitum for 42 d	Intestinal villus height ↑; Modulated gut microbiota. MDA ↓; CAT ↑	Reduced oxidative stress. Improved gut microbiota, amino acid metabolism	Polyphenol → intestine	Increased the average egg weight, albumen height, and eggshell thickness	[65]
Ellagic acid	Horses	Purity ≥ 90%, dietary admixture, 15, 30 mg/kg diet, ad libitum for 40 d	Modulated gut microbiota (Firmicutes, *Fibrobacter* ↑; *Pseudomonas* ↓); SCFAs ↑	Metabolized by gut microbiota to urolithins. Exerting microbiota-modulating activities	Polyphenol → intestine → growth performance	BW, ADG, ADFI ↑	[67]
Ellagic acid	Landrace × Yorkshire piglets	Purity ≥ 82%, dietary admixture, 0.1% diet, ad libitum for 14 d	Intestinal villus height ↑; Modulated gut microbiota (Ruminococcaceae, *Clostridium ramosum* ↑); SCFAs ↑; MDA ↓; GPx, CAT ↑	Reduced oxidative stress. Improved gut microbiota	Polyphenol → intestine → growth performance	ADG, ADFI ↑	[68]
Ellagic acid	Yili mares	Purity ≥ 90%, dietary admixture, 15, 30 mg/kg diet, ad libitum for 90 d	Modulated gut microbiota (Firmicutes ↑; Bacteroidetes, *Spirochaetes* ↓); SCFAs ↑; MDA ↓; SOD, CAT ↑; PRL, E2 ↑; GH, LH, PROG ↓	Reduced oxidative stress. Improved gut microbiota, lipid metabolism, carbohydrate metabolism	Polyphenol → intestine → Lactation performance	Lactation performance ↑	[69]
Ellagic acid	Arbor Acres broiler chickens	Purity ≥ 90%, dietary admixture, 75, 150, 300, 600 mg/kg diet, ad libitum for 28 d	Modulated gut microbiota (*Rothia*, *Neisseria*, *Actinomyces* ↑); SCFAs ↑; MDA ↓; GPx, SOD, CAT ↑	Reduced oxidative stress. Modulation of gut microbiota	Polyphenol → intestine → growth performance	BW, ADG, ADFI ↑	[70]
Resveratrol	Rat calvarial osteoblasts and bone marrow cells	0.05–2.5 μmol/L	Activated estrogen receptor ERα in bone tissue, induced the ER pathway, and activated the MAPK pathway	Promoted osteoblast proliferation and differentiation (late stage), promoted bone mineralization (ALP ↑)	Polyphenol → bone	/	[74]
Resveratrol	Mesenchymal stem cells	5 μmol/L	Inhibited RANKL ligand production, reduced bone resorption marker CTX, and enhanced bone marrow MSC differentiation into osteoblasts; Inhibited the RANKL/OPG/NF-κB pathway	Reduced bone resorption markers; enhanced bone marrow MSC osteogenic differentiation; improved lumbar and femoral BMD	Polyphenol → bone	/	[75]
Resveratrol	Weanling piglets	Purity ≥ 99%, dietary admixture, 300 mg/kg diet, ad libitum for 35 d	Enhanced intestinal integrity, Bacteroidetes ↑; Proteobacteria ↓; SCFAs ↑; Mediated Nrf2 pathway, regulated autophagy levels; IL-6, TNF-α ↓, ROS ↓	Alleviated intestinal inflammation and oxidative stress	Polyphenol → intestine	/	[76]
Resveratrol	Yellow-feathered broilers	Purity ≥ 99%, dietary admixture, 1,000–3,000 mg/kg diet, ad libitum for 14 d	Tight junction proteins (Occludin, claudin1) ↑; intestinal villus height ↑; SCFAs ↑; MDA ↓; GPx, SOD, CAT ↑	Reduced oxidative stress. Improved intestinal physiological function	Polyphenol → intestine → growth performance	BW, ADG, ADFI, Meat quality↑; FCR↓	[78]
Resveratrol	Simmental × Holstein F1 generation female calves	Purity ≥ 99%, dietary admixture, 2 g diet, ad libitum for 7 weeks	Modulated gut microbiota (*Parabacteroides*, *Christensenella*, and Ruminococcaceae ↑); SCFAs ↑; ROS ↓; CAT ↑; IL-1β ↓; IL-4 ↑	Reduced oxidative stress. Improved gut microbiota, tryptophan metabolism	Polyphenol → intestine → growth performance	BW, ADG, ADFI ↑	[79]
Curcumin	Yellow-feathered broilers	Purity ≥ 98%, dietary admixture, 200 mg/kg diet, ad libitum for 49 d	Tight junction proteins (ZO-1) ↑; Modulated gut microbiota (*Alistipes*, *Colidextribacter* ↑, *Faecalibacterium*, *Ligilactobacilli* ↓); SCFAs ↑; NF-κB, MyD88, TLR4 ↓; IL-10↑; Inhibited the NF-κB pathway	Reduced inflammation. Improved gut microbiota, lipid metabolism	Polyphenol → intestine → growth performance	BW, ADG, ADFI ↑; FCR ↓	[84]
Curcumin	Qingyuan partridge breeder hens	Purity ≥ 98%, dietary admixture, 100, 200, 400 mg/kg diet, ad libitum for 20 weeks	Tight junction proteins (ZO-1, occludin, claudin1) ↑; MUC2 ↑; Modulated gut microbiota (*Lactobacillus* ↑; *Barnesiella* ↓); SCFAs ↑; MDA ↓; GPx ↑; IL-22, TNF-α ↓	Reduced oxidative stress and inflammation. Improved gut microbiota, intestinal physiological function	Polyphenol → intestine → growth performance	BW, ADG, ADFI ↑; FCR ↓ (offspring chickens)	[85]

*ADG* Average daily gain, *ADFI* Average daily feed intake, *ALP* Alkaline phosphatase, *BW* Body weight, *FCR* Feed conversion ratio, *IFN-γ* Interferon gamma, *IGF-1* Insulin-like growth factor 1, *MCP-1* Monocyte chemoattractant protein-1, *MUC2* Mucin 2, *PER* Protein efficiency ratio, *PRL* Prolactin, *PROG* Progesterone, *TLR-4* Toll-like receptor 4, *VDR* Vitamin D receptor, *ZO-1* Zonula occludens-1, *SCFAs* Short-chain fatty acids, *ROS* Reactive oxygen species, *MDA* Malondialdehyde, *SOD* Superoxide dismutase, *GPx* Glutathione peroxidase, *CAT* Catalase, *Nrf2* Nuclear factor erythroid 2-related factor 2, *HO-1* Heme oxygenase-1, *ERK* Extracellular signal-regulated kinase, *JNK* c-Jun N-terminal kinase, *MAPK* Mitogen-activated protein kinase, *LPS* Lipopolysaccharide, *TLR4* Toll-like receptor 4, *NF-κB* Nuclear factor kappa B, *MyD88* Myeloid differentiation primary response 88, *TNF-α* Tumor necrosis factor-alpha, *IL* Interleukin

## Limitations and future perspectives

Although substantial evidence supports the beneficial effects of polyphenols on animal skeletal health through the gut-bone axis, several limitations should be considered. First, mechanistic evidence is predominantly derived from rodent models and in vitro studies, whereas direct validation in production animals remains relatively limited. Current studies involve diverse animal species, experimental conditions, and polyphenol preparations, resulting in heterogeneity that limits direct comparisons. Second, studies simultaneously evaluating gut microbiota, microbial metabolites, and bone phenotypes within the same animal model remain limited, making it difficult to fully establish causal relationships. Third, differences in polyphenol sources, doses, bioavailability, and metabolic transformations may influence their biological effects and practical applications. Therefore, further standardized studies are needed to clarify the mechanisms and improve the translation of polyphenol-based nutritional strategies into animal production. Improving the bioavailability of polyphenols represents an important future research direction, including optimizing the structure of natural polyphenols, developing novel delivery systems (nanoparticles, bioactive carriers, etc.), and combining them with other substances (probiotics, absorption enhancers, etc.) to enhance their efficacy.

## Conclusions

In summary, polyphenols can effectively remodel gut microbiota composition, enhance intestinal barrier integrity, promote the production of short-chain fatty acids, inhibit oxidative stress and inflammation, and maintain bone metabolic homeostasis and skeletal integrity by regulating endocrine-related pathways through the gut-bone axis. Furthermore, although different classes of polyphenols exhibit different regulatory mechanisms, existing evidence highlights their potential as functional feed additives to improve animal production performance.

## Data Availability

No datasets were generated or analysed during the current study.

## Abbreviations

ATF4: Activating transcription factor 4
BMD: Bone mineral density
BMP: Bone morphogenetic protein
BMSC: Bone marrow mesenchymal stem cell
CTX: C-terminal telopeptide of type I collagen
E2: Estradiol
EA: Ellagic acid
ER: Estrogen receptor
FAS: Fatty acid synthase
FOXO1: Forkhead box O1
FSH: Follicle-stimulating hormone
GH: Growth hormone
GLUT2: Glucose transporter type 2
HO-1: Heme oxygenase-1
LH: Luteinizing hormone
LPS: Lipopolysaccharide
MAPK: Mitogen-activated protein kinase
MDA: Malondialdehyde
NF-κB: Nuclear factor kappa B
NLRP3: NLR family pyrin domain-containing protein 3
Nrf2: Nuclear factor erythroid 2-related factor 2
OPG: Osteoprotegerin
PEPT1: Peptide transporter 1
RANK: Receptor activator of nuclear factor kappa-B
RANKL: Receptor activator of nuclear factor kappa-B ligand
RES: Resveratrol
ROS: Reactive oxygen species
SCFAs: Short-chain fatty acids
SOD: Superoxide dismutase
TLR4: Toll-like receptor 4
TPH-1: Tryptophan hydroxylase 1
